# Atomistic insights into the anisotropic mechanical properties and role of ripples on the thermal expansion of h-BCN monolayers†

**DOI:** 10.1039/c8ra08076c

**Published:** 2019-01-09

**Authors:** Siby Thomas, Sang Uck Lee

**Affiliations:** Department of Bionano Technology, Hanyang University Ansan 15588 Korea sulee@hanyang.ac.kr; Department of Chemical & Molecular Engineering, Hanyang University Ansan 15588 Korea

## Abstract

Monolayer boron–carbon–nitrogen (h-BCN) has been studied in comparison with graphene and hexagonal boron nitride (h-BN) using classical molecular dynamics (MD) simulations with an aim to better understand the structural and thermal behaviors and the anisotropic mechanical properties. The structural features of the simulated sample were analyzed using the pair-correlation function and a full width at half maximum (FWHM). As a hetero-structure of h-BN and graphene, the C–C bond in the h-BCN is responsible for an improved FWHM compared to graphene. Consistent with graphene and h-BN, the in-plane lattice parameter of h-BCN shows thermal contraction over a wide range of temperatures and exhibits a system size dependence. The observed thermal contraction is explained by the presence of out-of-plane bending modes excited at finite temperatures. A tensile test has been performed as a suitable means of measuring the mechanical properties of the h-BCN sheet for zigzag and armchair orientations and found that it is mechanically anisotropic and stable under various strain directions and temperatures. The fracture strength of h-BCN is affected by loading direction and temperature. We found that the Young's modulus of h-BCN is smaller than that of graphene but is higher than that of an h-BN monolayer, suggesting that h-BCN has high mechanical stiffness. Our modeling-based findings provide a guide for future experiments concerning the physical properties of this advanced composite material.

## Abstract 2

2D h-BCN is a novel planar semi-conducting material akin to graphene and h-BN with high thermal mechanical stability suitable for the design of h-BCN-based composite materials.

## Graphical abstract

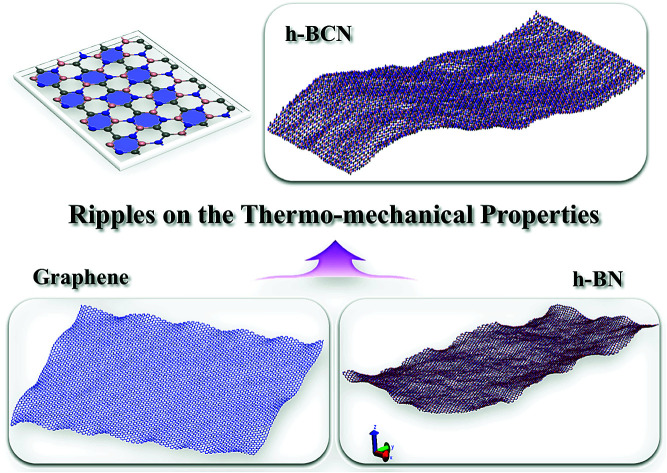

## Introduction

Due to the success of graphene,^1,2^ alternative layered and non-layered two-dimensional (2D) materials have become a center of interest for potent research due to their unique chemical and physical properties. Graphene-analogous materials have a wide range of applications for electrochemical storage devices and optoelectronic devices due to their unusual features. This 2D materials family has unique anisotropic mechanical, optical, electrical, and thermal properties which create promising opportunities for many applications, including the design of novel devices^3–6^ and aerospace components. Hexagonal boron nitride (h-BN) is one of the well-studied graphene-analogous materials.^7,8^ The atomic lattice of h-BN consists of a honeycomb structure with sp^2^ hybridization and possesses high bond strength. Even though the physical properties of h-BN are comparable to graphene, it is a wide-bandgap insulator with an electronic band-gap of 5.97 eV,^7^ whereas graphene is a zero-band-gap material. However, the application of graphene in nanoelectronics is limited because it does not exhibit a bandgap and is typically categorized as a semi-metallic material. The very high bandgap with insulating characteristics of h-BN also restrict its application in optoelectronics or nanoelectronics.

Inspired by the outstanding features of graphene and h-BN, efforts have been made to study additional hexagonal structures composed of nitrogen, boron, and carbon atoms. Most recently, Beniwal *et al.* reported the successful experimental synthesis of a 2D graphenic with a ternary monolayer containing boron, carbon, and nitrogen atoms; they named this material “monolayer boron-carbon-nitrogen” (h-BCN).^9^ By using density functional theory (DFT) calculations, they predicted that the direct electronic band gap of h-BCN is 1.5 eV and is midway between that of gapless graphene and that of insulating h-BN.^9^ In addition to the successful synthesis and characterization of h-BCN, the possible synthesis of other graphitic carbon nitride materials has been reported recently.^10,11^

By employing first-principles DFT calculations, Jiao *et al.* explored the structural stability, electronic properties, mechanical stability, and optical properties of different types of monolayer BC_2_N.^12^ Mortazavi *et al.* performed extensive MD simulations on graphene like C_3_N and reported superior mechanical properties and thermal conductivity comparable to graphene.^13^ Using MD simulations, Lin *et al.* studied the anisotropic thermal transport properties in monolayer BC_2_N.^14^ Most recently, Zhang *et al.* examined the thermal conductivity in the h-BCN monolayer using MD simulations. They reported a significantly lower thermal conductivity for h-BCN in comparison with graphene and h-BN.^15^ Using classical MD simulations, we performed original studies on h-BCN to determine the following: the structural stability, the effect of ripples on the thermal expansion properties, and the anisotropic mechanical stability as a function of the temperature. To the best of our knowledge, there have been no previous theoretical or experimental investigations of these properties. Therefore, predictions based on our computation results can serve as a guide to researchers interested in the various properties of this novel 2D material.

## Computational details

Various properties of the h-BCN monolayer are studied using classical MD simulations that performed using the LAMMPS^16^ package. Simulation boxes with a total number of 9600 atoms were used in most of the MD simulation. In this work, a Tersoff type empirical inter-atomic potential reported in Kinaci *et al.*^17^ parameterised by Sevik *et al.*^18^ and Lindsay *et al.*^19^ has been used to model the interaction between the boron–carbon–nitrogen atoms in the h-BCN monolayer. We have studied the properties of the system by varying temperatures between 100 K and 1000 K. All the simulations were initiated by relaxing the atomistic configuration of the h-BCN system to a minimum potential energy state. In the first phase of the simulations, we relaxed the h-BCN nanosheet under isothermal–isobaric ensemble (NPT) ensemble for 200 picoseconds (ps) with a time step of 0.001 ps. The simulation temperature and pressure were controlled using a Nose–Hoover thermostat algorithm and a barostat algorithm, respectively. We applied periodic boundary conditions in all three dimensions, and we kept the direction of thickness along the *Z*-direction as high to ensure that only a single sheet of h-BCN was involved in the simulation procedure. During the MD study of the h-BCN structure, we solved the equations of motion *via* the Velocity-Verlet scheme. After an NPT run of 100,000 steps (a time range of 100 ps), we performed an MD run with the canonical ensemble (NVT) for another 100 000 steps to further equilibrate the system at each temperature.

During the stress–strain calculations, in order to avoid spurious behavior when predicting the mechanical properties using optimized Tersoff interatomic potential, we modified the lower cut-off value of the potential to 2.10 Å. After ensuring proper equilibration and relaxation using NPT ensemble for 200 ps, we applied uniaxial tension by pulling the sheet along the *x*-direction (zigzag) or along *y*-direction (armchair) at a strain rate of 0.001 per picoseconds, while in the direction perpendicular to the loading direction, the traction force was free. To maintain the desired boundary conditions, we enforced the NPT along all directions. We performed the simulations for 500 picoseconds. Stress calculations were performed with virial formulation using the following equation:1
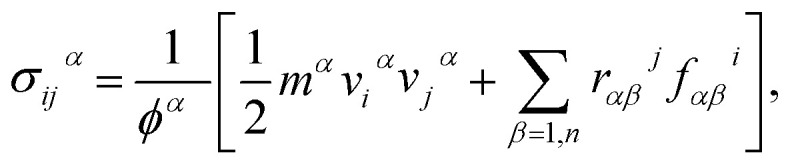
where *i* and *j* denote indices in the Cartesian coordinate system; *α* and *β* are the atomic indices; *m*^*α*^ and *v*^*α*^ are the mass and velocity of atom *α*; *r*_*αβ*_ is the distance between atoms *α* and *β*, *ϕ* is the atomistic volume of the system, and *f*_*αβ*_^*i*^ is the force along direction *i* on atom *α* due to atom *β*.

## Results and discussion

### A Structural stability

**Fig. 1:**
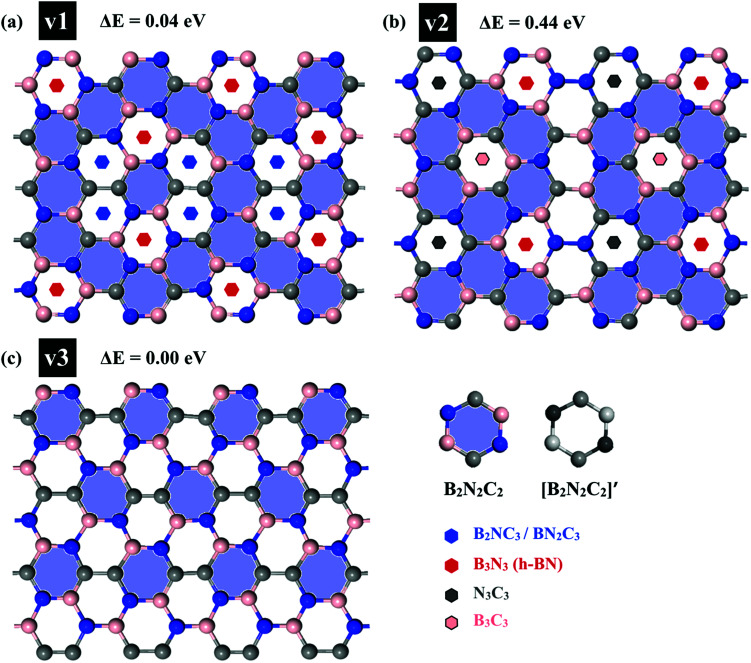
Three possible h-BCN monolayers structures, namely (a) h-BCN_v1, (b) h-BCN_v2 and (c) h-BCN_v3 with relative stability.

**Fig. 2:**
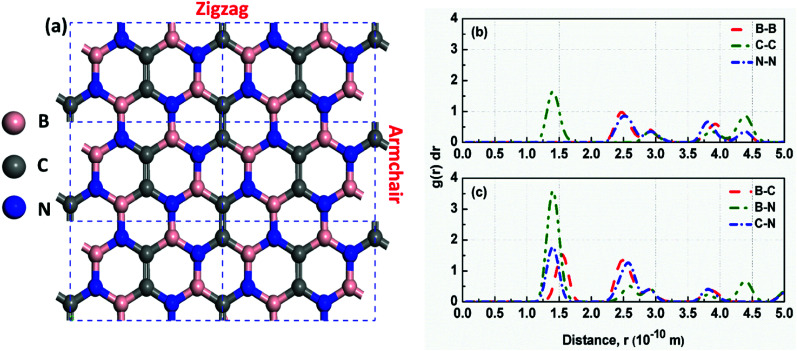
(a) The optimized structure of h-BCN_v3 monolayer. The dashed black rectangles represent the unit cell of h-BCN with a lattice constant of 7.59 Å; (b) and (c) Radial distribution function of B–B, C–C, N–N, B–C, B–N and C–N pairs in a h-BCN sheet at 300 K. The value of RDF at any *r* decreases as the temperature increases due to thermal broadening.

First, we examined the possible h-BCN monolayer stoichiometry as reported in the experimental work of Beniwal *et al.*^9^ In our atomistic studies herein, we also noticed that h-BCN has three possible structures (h-BCN_v1, h-BCN_v2 and h-BCN_v3) with different coordination environments around the B, N, and C atoms of the networks within the same B_2_C_2_N_2_ stoichiometry. Our cohesive energy calculation shows that the h-BCN_v3 model possesses the highest structural stability, followed by h-BCN_v1 and h-BCN_v2, as shown in Fig. 1. The three h-BCN monolayers are composed of B_2_C_2_N_2_ units, but the orientation and arrangement of B_2_C_2_N_2_ units are different for each model. Therefore, they have different connectivity between the B_2_C_2_N_2_ units and different types of hexagonal units: B_2_NC_3_, BN_2_C_3_, B_2_C_2_N_2_, B_3_N_3_, N_3_C_3_, and B_3_C_3_. In contrast, while the h-BCN_v1 and h-BCN_v3 models have C–C and B–N connecting bonds between the B_2_C_2_N_2_ units, the h-BCN_v2 model has a B–C connecting bond. Comparing the bond length of the connecting bonds, we found that the B–C (1.52 Å) bond is longer than both the C–C (1.42 Å) and B–N (1.45 Å) bonds. The weak B–C connecting bond makes the h-BCN_v2 model unstable. In addition, h-BNC_v2 has a B_3_C_3_ unit composed of only a weak B–C bond. Although both h-BCN_v1 and h-BCN_v3 models have the same C–C and B–N connecting bonds, the different arrangement of their B_2_C_2_N_2_ units leads to varying contributions of C–C and B–N bonds to the material's structural stability. The h-BCN_v1 model is more stable than the h-BCN_v2 model due to the greater contribution of the C–C bond. Since the h-BCN_v3 model possesses the most energetic stability of the three models, we focus the remaining discussion on the MD simulation with the h-BCN_v3 configuration. Hereafter, h-BCN_v3 is denoted simply as h-BCN. Fig. 2a shows a representative structure of the h-BCN monolayer.

Analysis of the structural properties of h-BCN was carried out at various temperatures. The radial distribution function (RDF) (or equivalently, the pair correlation function), is a measure that can be used to forecast the stability of the atomistic structure. To evaluate the structural stability of h-BCN nanosheet, the RDF was used. The RDF is a Dirac delta function at zero kelvin temperature due to the fact that there exists a well-defined separation between atom pairs in any perfect crystal. But the delta functions broaden into smooth peaks at high temperatures due to thermal vibrations. The width of the RDF peaks is proportional to the root mean squared displacement of the atoms from their equilibrium position. As the temperature increases, the height of peaks in the RDF decreases due to thermal broadening. The RDFs of h-BCN for all the possible atomic distances at 300 K are shown in Fig. 2b and c. During MD simulation, the full width half maximum (FWHM) of RDF is considered to be intimately related to the integrity of the atomistic structure. We predicted that the structures with sharp peaks and relatively smaller values of FWHM for RDF would possess better structural integrity. We also studied the stability and structural integrity of h-BCN nanosheet at different temperatures with the help of the FWHM values estimated using the RDF, as shown in Fig. S1.† The calculated FWHM values for various atomic distances of h-BCN at a temperature of 300 K is shown in Table 1. Compared to the C–N bond (1.42 Å), the C–B bond (1.52 Å) is weaker and is highly sensitive to temperature. From Fig. S1,† it is clear that B–B and B–C show a large increase in FWHM around 1000 K. Moreover, the absence of a chemical bond in B–B is responsible for the bond weakening. Compared to the C–C atomic distance of graphene, a lower FWHM value of h-BN and h-BCN implies that the structure of h-BCN is robust and stable possessing better structural integrity with respect to the empirically observed interatomic potential. It is also worth noting that the FWHM of h-BCN shows a similar trend to that of h-BN. Even though h-BCN, h-BN, and graphene have similar hexagonal lattice structures, the different atom constituents lead to visible changes in the structural and energetic stability. In addition to this, the C–C bond in the h-BCN hetero-structure is responsible for an improved FWHM compared to graphene. A similar pattern of structural robustness was reported for hydrogenated h-BN nanotubes^20^ and h-BN nanosheet.^21,22^

**Table 1:** FWHM for various atomic distances of monolayer h-BCN at 300 K in comparison with graphene and h-BN

	FWHM (Å)					
	B–B	C–C	N–N	B–C	B–N	C–N
h-BCN	0.0954	0.0910	0.0980	0.0854	0.0855	0.0600
h-BN	0.0881		0.0954		0.0897	
Graphene		0.1863				

### B Linear thermal expansion coefficient

**Fig. 3:**
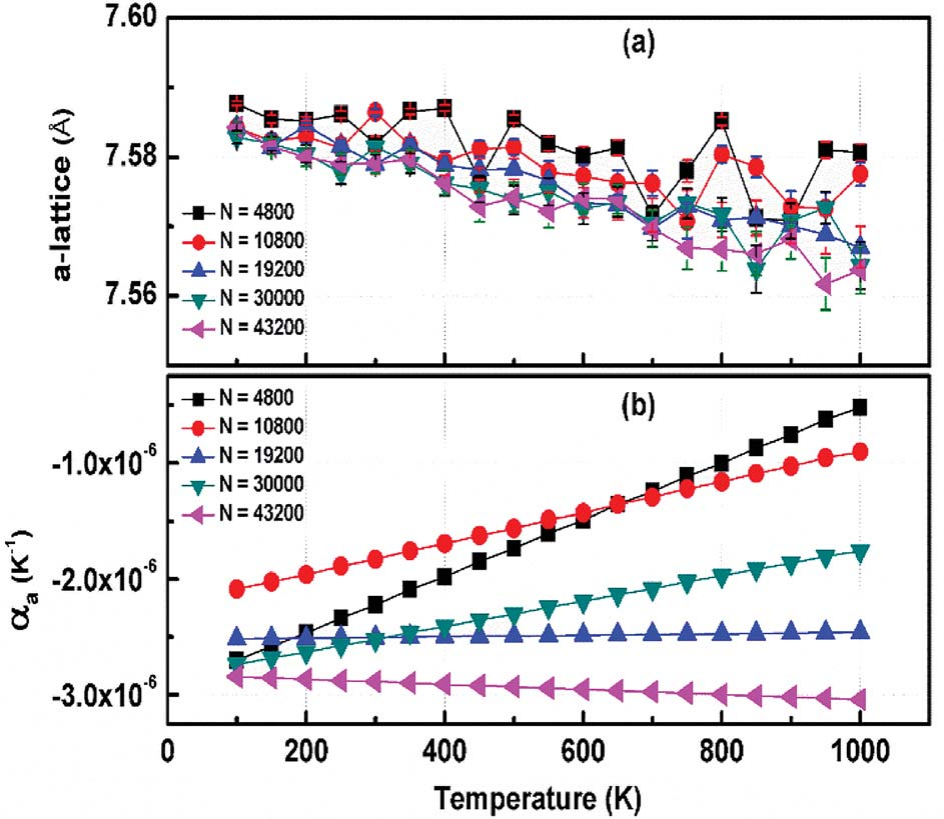
(a) The temperature dependence of the in-plane lattice parameter (*a*-lattice) of 2D h-BCN; (b) the linear thermal expansion coefficients (LTECs) as a function of temperature.

The structural stability of 2D atomic crystals and surfaces have been a point of disagreement in the theory of condensed matter. Based on the Mermin–Wagner theorem,^23^ which states that long wavelength fluctuations destroy the long-range order for 2D crystals. Similarly, 2D membranes embedded in a 3D space tend to be rippled. However, these dangerous fluctuations can result in a two-dimensional membrane, and exhibit strong height fluctuations. But in the case of graphene and 2D h-BN, the repression of long wavelength ripples takes place *via* a strong anharmonic coupling between the in-plane stretching and the out-of-plane bending modes. The height fluctuations on the surface are known as ripples,^24^ and they essentially stabilize these 2D membranes.^25,26^ We expected similar out-of-plane excursions in h-BCN sheet. One advantage of classical MD simulations is that they can include the full anharmonicity of the inter-atomic potential. Another advantage is that the simulations can include millions of atoms. We studied the change of the in-plane lattice parameter and the linear thermal expansion coefficient (LTEC) as functions of temperature. To understand the role of ripples in the thermal expansion properties of 2D h- BCN, simulations were done with a 2D h-BCN sheet consisting of various cell sizes. We used simulation cells with 4800, 10 800, 19 200, 30 000, and 43 200 atoms to include the effect of long wavelength fluctuations. Fig. 3a and b display the temperature dependence of the in-plane lattice parameter and the LTEC. We found that the lattice parameter decreases as temperature increases. The LTEC is computed by the direct numerical differentiation of the lattice constant *a* using the equation:2
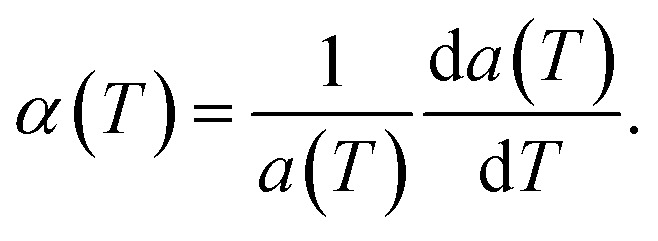


The LTEC is negative in the entire computed temperature interval up to 1000 K. This is consistent with the case of graphene and h-BN, as tabulated in Table 2. We also found that the temperature evolution of the *a*-lattice is system size-dependent (Fig. 2). This system size dependence of the *a*-lattice is due to the existence of large-scale ripples^24,32^ in h-BCN, which is similar to graphene and h-BN and in the actual physical context, which cannot be fitted adequately inside the small simulation cells.^33^ The high thermal stability of h-BCN can be effectively utilized in the manufacturing of h-BCN composite materials. Knowledge of how h-BCN composite materials react to high temperatures is vital to optimizing the manufacturing process. The negative thermal expansion coefficient of h-BCN composites may reduce the thermal stresses and weaken the bonds as temperature rises.

**Table 2:** The system size dependence of linear thermal expansion coefficients (LTECs) of h-BCN_v3 at 300 K in comparison with graphene and h-BN. The obtained LTECs are all negative and show a system size dependence. Please note that the total number of atoms present in graphene and h-BN are approximately similar to the present study, not exactly. The values given in bracket represents the experimentally obtained results

Number of atoms	LTEC (×10^−6^ K^−1^)		
	Graphene	h-BN	h-BCN
4800	−4.100 (ref. 26) (−5.500,^27^ −7.000,^28,29^ −8.000 (ref. 30))	−5.151 (ref. 26)	−2.224
10 800	−4.380 (ref. 26)	−5.508 (ref. 26)	−1.828
19 200	−4.350 (ref. 26)	−3.000,^25,31^ −5.509 (ref. 26)	−2.504
30 000	—	—	−2.522
43 200	−4.524 (ref. 26)	−5.670 (ref. 26)	−2.887

### C Evaluation of the anisotropy in the elastic constants

**Fig. 4:**
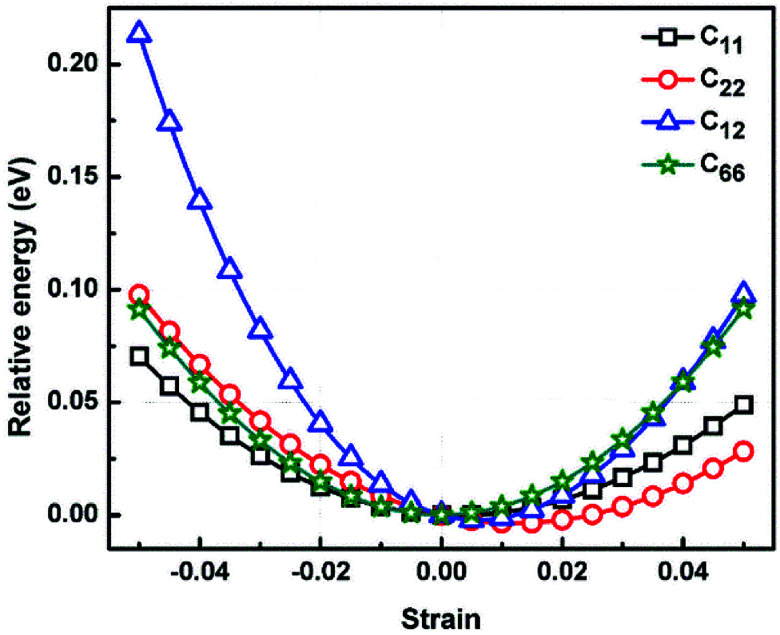
The relative energy *versus* strain response graph of h-BCN sheet using molecular statics simulations for the analysis of elastic constants. The relative energy is plotted as a function of various specific strains necessary for calculating the elastic tensors.

This section details the results of the elastic constants of the h-BCN sheet at zero kelvin temperature, which we computed utilizing molecular statics simulations. We have carried out calculations pertaining to an h-BCN sheet of infinite spatial extent along the *x*- and *y*-axes, which correspond to the zigzag and armchair directions, as shown in Fig. 2a. A detailed description of the calculation procedure can be found in our previous work.^34^ We evaluated the elastic behavior of h-BCN sheet by employing the energy–strain approach. The mathematical explanation of the in-plane interactions of the matrix of a second-order elastic constant is denoted as:3
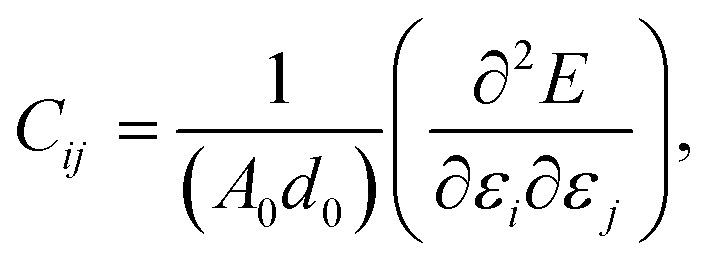
where, *E*, *A*_0_, *d*_0_, and *ε* are the equilibrium energy, effective area and the effective thickness (the van der Waals distance), and the strain tensor associated with the calculation. The elastic energy per unit area *E*(*ε*) can be expressed using the polynomial form: 
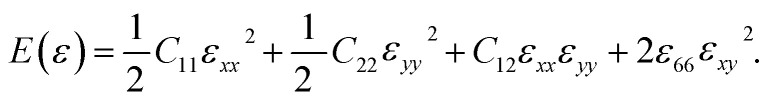
 We calculated the four non-zero 2D elastic constants: *C*_11_, *C*_22_, *C*_12_ and *C*_66_ (1-*xx*, 2-*yy*, 6-*xy* in the standard Voigt notation) of the h-BCN sheet by fitting the strain–relative energy curve, as shown in Fig. 4. The Young's modulus along the *x* (*E*_*x*_) and *y* (*E*_*y*_) directions can be derived from the computed values of elastic constants using the equations: 
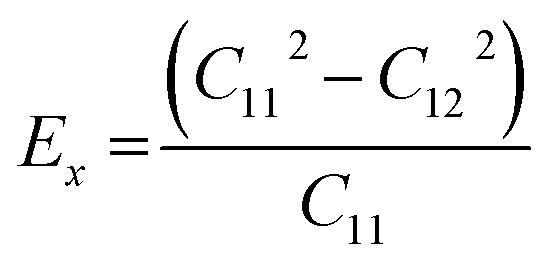
 and 
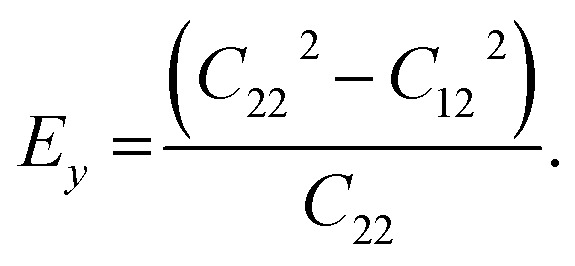
 The calculated values of Young's modulus at zero kelvin using molecular statics calculations are 786 GPa and 830 GPa respectively along the *x* and *y* directions. The different values of *E*_*x*_ and *E*_*y*_ indicate that h-BCN sheet is mechanically anisotropic. From the computed elastic constants, we also deduced the directional dependent Poisson's ratios as 
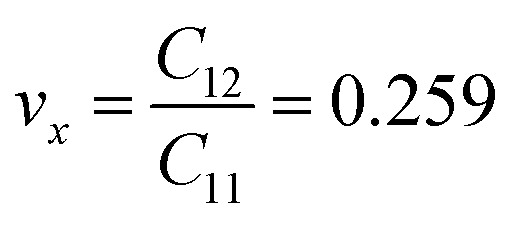
 and 
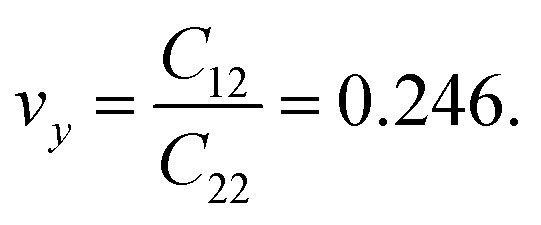
 Although the calculated Young's modulus of h-BCN is smaller than that of graphene^22,35^ (*E*_*a*_ = *E*_*b*_ = 939 ± 10 GPa), they are relatively higher compared to that of the h-BN monolayer^31,34^ (*E*_*a*_ = *E*_*b*_ = 750 ± 30 GPa), suggesting that h-BCN has good mechanical properties with high anisotropy. The highly anisotropic characteristic of h-BCN is attributed to its structural morphology. It has also been observed that h-BCN possess higher stiffness in comparison to other 2D materials such as monolayer SiC,^36^ C_3_N,^13^ and pentagraphene.^37^ This suggests that h-BCN has potential use in the fields of transportation, aerospace, power generation and energy storage. In our study, the anisotropic behavior is clearly observed from the direction dependent elastic constants, and the h-BCN sheet also satisfies Born's mechanical stability criterion.

### D Mechanical fracture behavior

**Fig. 5:**
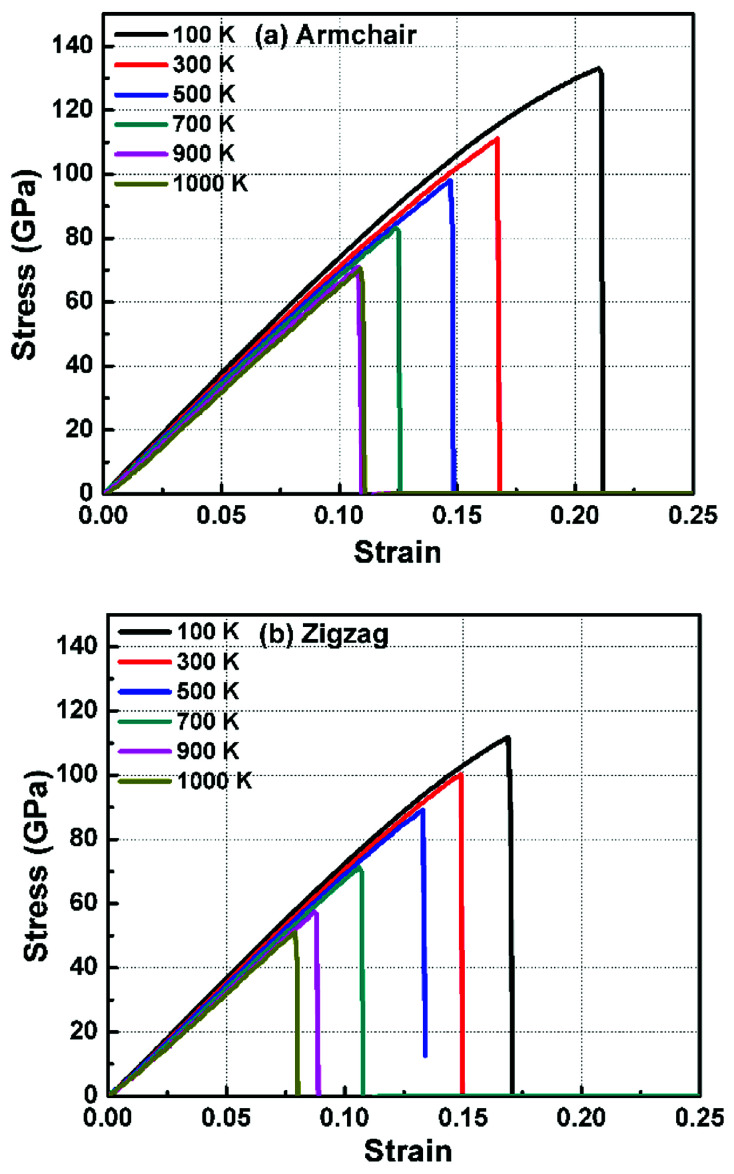
The Illustration of the temperature dependence of the stress–strain response of h-BCN sheet under uniaxial tension along (a) armchair and (b) zigzag direction at a strain rate of 0.001 ps^−1^.

In order to study the fracture behavior of h-BCN nanosheet, simulations were carried out using an optimized cut-off function of the interatomic potential. During the initial stages of stress–strain analysis, we carried out the relaxation for 200 ps in the NPT ensemble. The change in temperature and potential energy of the h-BCN system during the relaxation is shown in Fig. S2.† Even though we performed relaxation for 200 ps, we observed that the system reaches a properly relaxed state after 50 ps, as shown in Fig. S2a and b.† After the relaxation, we applied deformation by pulling the h-BCN sheet along the *x*-direction (zigzag) or the *y*-direction (armchair) with temperatures ranging from 100 to 1000 K, in conjunction with three different strain rates: 0.0001, 0.001 and 0.005 ps^−1^. We observed that this material possesses strong anisotropic characteristics that depend on numerous factors, including temperature, strain rate, and loading direction. MD simulations were performed to investigate the effect of strain rate and temperature on the mechanical responses of h-BCN sheet. We monitored the engineering stress–strain response of h-BCN within a temperature range of 100–1000 K, under tension load along both armchair and zigzag directions. The observed anisotropic stress–strain response along the armchair and zigzag directions are shown in Fig. 5a and b, respectively. The fracture strength (engineering stress) and the strain decrease over the studied temperature range along both the armchair and zigzag directions. At 300 K, the observed fracture strength is 111 GPa along the armchair direction and 100 GPa along the zigzag direction with a strain rate of 0.001 ps^−1^. The corresponding fracture strains are 0.17 and 0.15 respectively along the armchair and zigzag direction. The lower ultimate tensile strain and stress for the zigzag orientation is likely due to the deformation of bonds, which are directly subjected to the load at the beginning of the elongation process, whereas in the armchair situation the external loads are weakened through both bond elongation and bond angle variation. We also observed that the armchair deformations exhibit a mechanically strengthening behavior in the stress–strain evolution process. This can be explained on the basis of the spatial bond distributions and bond breakage mechanism, which suggest that more ductile materials may exhibit such features during the initial stages of the stress–strain evolution procedure.^38^

**Fig. 6:**
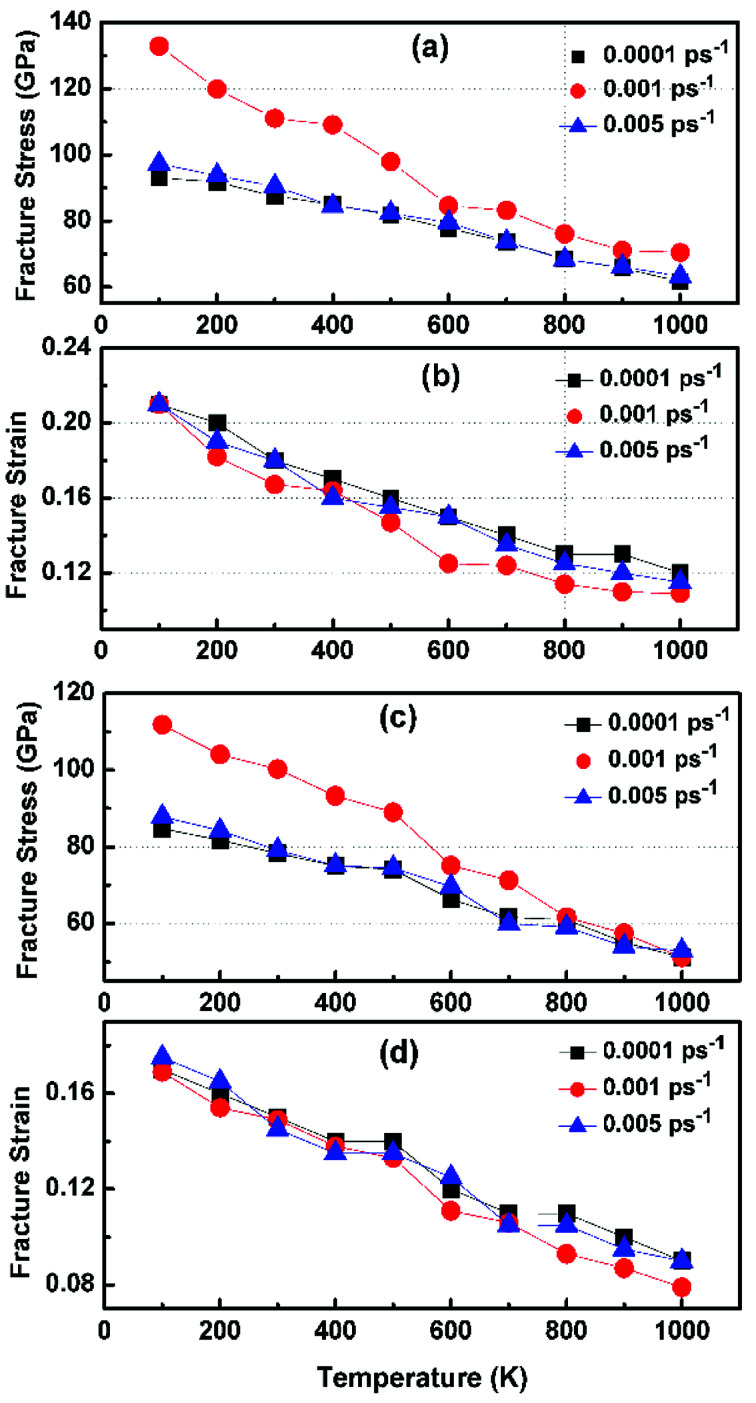
Variation of fracture stress and fracture strain with temperature in the (a and b) armchair and (c and d) zigzag directions. The simulations are performed with temperatures ranging from 100 to 1000 K in conjunction with three different strain rates of 0.0001, 0.001 and 0.005 ps^−1^.

**Fig. 7:**
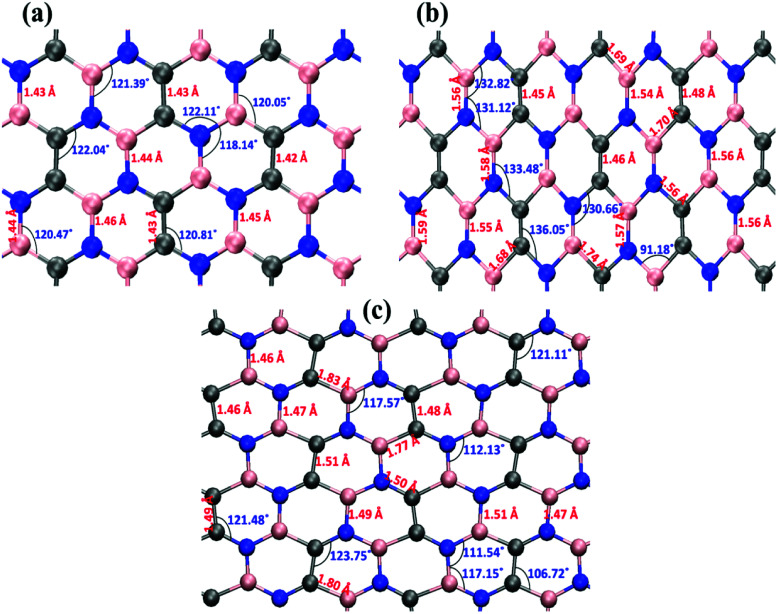
The bond length and bond angle distributions for the (a) unstrained, (b) strained in the armchair direction and (c) strained in the zigzag direction configurations. Considerable change in the bond length and bond angle has been observed after applying a deformation either in the armchair direction or in the zigzag direction.

Fig. 6 illustrates the stress–strain responses of the tensile properties of the h-BCN sheet at different strain rates and temperatures. We performed the simulations with temperatures ranging from 100 to 1000 K, in conjunction with three different strain rates (0.0001, 0.001, and 0.005 ps^−1^) along the armchair direction (Fig. 6a and b) and the zigzag direction (Fig. 6c and d). The maximum stress in both armchair and zigzag directions occurs at a strain rate of 0.001 ps^−1^. The lack of proper experimental evidence limits any direct comparisons of the obtained stress–strain response of h-BCN with that of other materials. However, our approach is qualitatively in agreement with both the experimental and theoretical investigations of graphene and h-BN.^39–43^ This qualitative agreement substantiates the validity of our numerical model. From our stress–strain investigations, we concluded that the failure morphology of h-BCN sheet is dependent on the strain rate.^44^ We also studied the failure morphology of h-BCN upon tensile loading. In most materials, the fracture mechanism is affected by temperature and strain rate. Due to h-BCN's atomically thin nature, it easily undergoes various types of fracture mechanisms and deformations, despite its high stiffness. Bond length and bond angle of both the pristine h-BCN sheet and the strained h-BCN sheet were studied, with varying orientations. Fig. 7a shows the calculated bond length and bond angles of an unstrained pristine h-BCN sheet. We observed considerable changes in bond length and bond angle after applying a deformation in the armchair and zigzag directions, as shown in Fig. 7b and c respectively.

The structural details of an individual atomic network determine the material's strength in any particular direction. As we subjected the h-BCN network to uniaxial strain, its response was manifested by changes in the bond angles as well as the bond lengths. At a completely relaxed zero strain state, we observed a single bond angle around 120° in the angle distributions of one hexagonal ring in the h-BCN (Fig. 7a). This configuration corresponds to the most unrestrained geometry of the sp^2^ hybridization of the atoms in the BCN network. As soon as we applied strain to the system, it deformed by changing the bond lengths and angles. The strained configurations along the armchair and zigzag directions are shown in Fig. 7b and c, respectively. For each case, we observed a notable difference between the armchair and zigzag directions in terms of their bond and angle distributions. The widened angle is higher in the armchair case than in the zigzag case. A similar pattern of bond angles and lengths has been reported in the case of other 2D materials such as biphenylene, phagraphene and nitrogenated holey graphene.^45^ We attribute the high stretchability along the armchair direction to the significant widening of the bond lengths and angles in the h-BCN system. We also found that certain stages of deformation in h-BCN involve the formation of defects, cracks, and the breaking of bonds and that the edges of the h-BCN material are easily affected by the deformation, as shown in Fig. S3 and S4.†

**Fig. 8:**
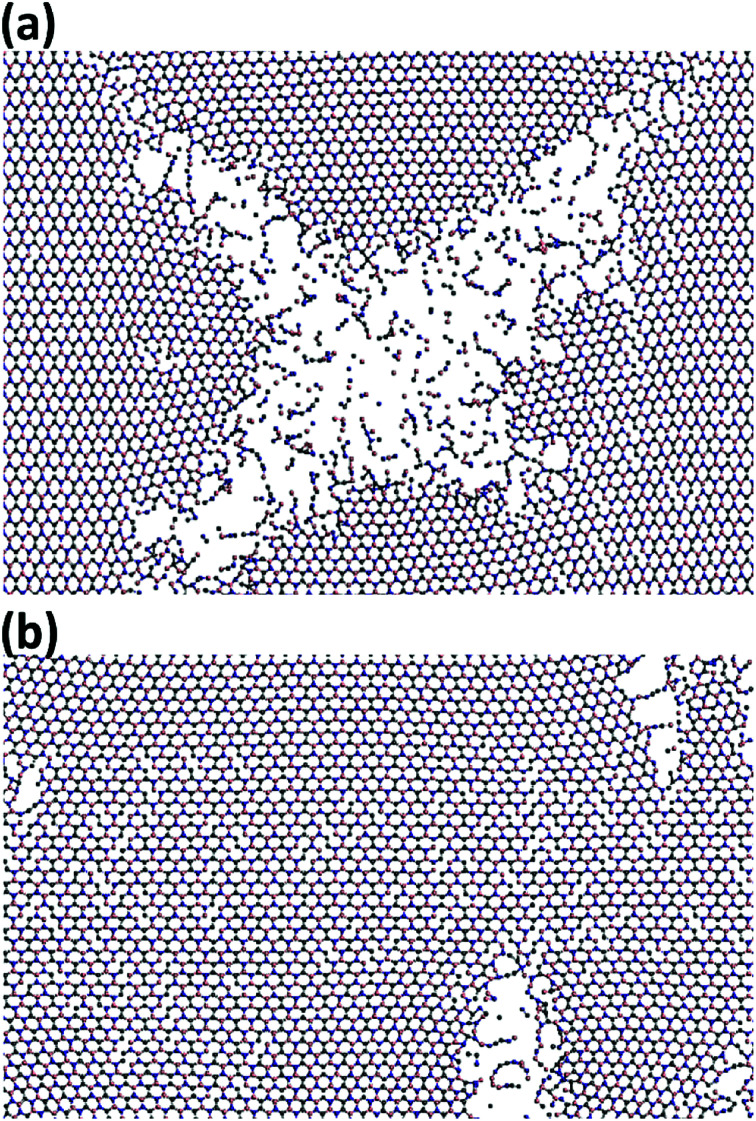
Observed ductile fracture patterns during the uniaxial deformation of h-BCN monolayer along the (a) armchair and (b) zigzag orientations at 300 K.

In Fig. 8, the failure process of h-BCN sheet along armchair and zigzag directions at 300 K are depicted. During the initial stages of tensile loading, bond elongation followed by bond breaking happening in both armchair and zigzag orientations, as shown in Fig. S3 and S4.† Further increasing the strain level along the armchair and zigzag directions lead to more bond breakage. The formation of chain type rupture in the sheet indicates the ductile nature of mechanical failure for h-BCN sheet. Unlike pristine graphene, the bond breaking and failure process happen at distinctly different strain levels in h-BCN sheet, which also infers a ductile failure process in the material. The ductile failure behavior predicted by the Tersoff potential is in good agreement with the earlier atomistic studies of pristine and defective graphene,^39,40^ graphene-like C_3_N,^13^ boron nitride nanosheets,^46,47^ and phagraphene^48^ using the Tersoff-like potential. Based on our calculations we found that h-BCN monolayer shows a higher ductile failure along the armchair direction when compared to the zigzag direction. This can be confirmed by the obtained failure strain values and is found to be 0.17 and 0.15 respectively along the armchair and zigzag direction at 300 K. In the present work, we have focused on the mechanical stability analysis of pristine monolayer BCN. We are of the opinion that incorporating defects in the computational studies could provide more realistic picture prior to experimental analysis. In view of this, a systematic study of the thermomechanical properties of defective monolayer BCN would also be worthwhile.

## Conclusion

In this study, we performed classical MD simulations based on the optimized Tersoff potential to investigate the thermo-mechanical properties of an h-BCN monolayer. We examined the effects of temperature, system size, and tensile properties on h-BCN. Our major findings are as follows: (1) the narrow FWHM of h-BCN as compared to graphene is attributed to the better structural integrity of h-BCN with respect to the used empirical interatomic potential which leads to a lesser spread in the interatomic distances. The C–C bond in the h-BCN hetero-structure is also responsible for an improved FWHM compared to graphene. (2) At finite temperatures, a thermal contraction is observed and is due to the presence of out-of-plane bending modes excited, along with thermal energy which reduce the thermal stresses and weakening the bonds as temperature rises which guarantee the durability of h-BCN based composite materials for better practical applications. (3) Akin to graphene and h-BN in the hexagonal lattice, the calculated Young's modulus of h-BCN is smaller than that of graphene, but is higher than that of h-BN monolayer, suggesting that h-BCN has good mechanical properties with high anisotropy. (4) The elastic moduli of h-BCN decreases with temperature and is affected by the loading direction. (5) Finally, the lower ultimate tensile strain and stress for the zigzag orientation is likely due to the bonds deformations, which are directly subjected to the load at the beginning of the elongation process, whereas in the armchair situation the external loads can are more spread out *via* both bond elongation and bond angle variation. We believe that the results we obtained may help in the design of h-BCN-based composite materials for large-scale applications.

## Conflicts of interest

There are no conflicts to declare.

## Supplementary Material

RA-009-C8RA08076C-s001

## References

[cit1] Novoselov K. S., Geim A. K., Morozov S. V., Jiang D., Zhang Y., Dubonos S. V., Grigorieva I. V., Firsov A. A. (2004). Science.

[cit2] Geim A. K., Novoselov K. S. (2007). Nat. Mater..

[cit3] Yang S. X., Yang Y. H., Wu M. H., Hu C. G., Shen W. F., Gong Y. J., Huang L., Jiang C. B., Zhang Y. Z., Ajayan P. M. (2018). Adv. Funct. Mater..

[cit4] Liu E. F., Fu Y. J., Wang Y. J., Feng Y. Q., Liu H. M., Wan X. G., Zhou W., Wang B. G., Shao L. B., Ho C. H., Huang Y. S., Cao Z. Y., Wang L. G., Li A. D., Zeng J. W., Song F. Q., Wang X. R., Shi Y., Yuan H. T., Hwang H. Y., Cui Y., Miao F., Xing D. Y. (2015). Nat. Commun..

[cit5] Yan J., Hu M., Li D., He Y., Zhao R., Jiang X. Y., Song S. P., Wang L. H., Fan C. H. (2008). Nano Res..

[cit6] Yang H., Jussila H., Autere A., Komsa H. P., Ye G. J., Chen X. H., Hasan T., Sun Z. P. (2017). ACS Photonics.

[cit7] Watanabe K., Taniguchi T., Kanda H. (2004). Nat. Mater..

[cit8] Park J. H., Park J. C., Yun S. J., Kim H., Luong D. H., Kim S. M., Choi S. H., Yang W., Kong J., Kim K. K., Lee Y. H. (2014). ACS Nano.

[cit9] Beniwal S., Hooper J., Miller D. P., Costa P. S., Chen G., Liu S. Y., Dowben P. A., Sykes E. C. H., Zurek E., Enders A. (2017). ACS Nano.

[cit10] Thomas A., Fischer A., Goettmann F., Antonietti M., Muller J. O., Schlogl R., Carlsson J. M. (2008). J. Mater. Chem..

[cit11] Mahmood J., Lee E. K., Jung M., Shin D., Jeon I. Y., Jung S. M., Choi H. J., Seo J. M., Bae S. Y., Sohn S. D., Park N., Oh J. H., Shin H. J., Baek J. B. (2015). Nat. Commun..

[cit12] Jiao L. N., Hu M., Peng Y. S., Luo Y. T., Li C. M., Chen Z. G. (2016). J. Solid State Chem..

[cit13] Mortazavi B. (2017). Carbon.

[cit14] Lin C. P., Zhang X. L., Rao Z. H. (2017). Nano Energy.

[cit15] Zhang Y. Y., Pei Q. X., Liu H. Y., Wei N. (2017). Phys. Chem. Chem. Phys..

[cit16] Plimpton S. (1995). J. Comput. Phys..

[cit17] Kinaci A., Haskins J. B., Sevik C., Cagin T. (2012). Phys. Rev. B: Condens. Matter Mater. Phys..

[cit18] Sevik C., Kinaci A., Haskins J. B., Cagin T. (2011). Phys. Rev. B: Condens. Matter Mater. Phys..

[cit19] Lindsay L., Broido D. A. (2010). Phys. Rev. B: Condens. Matter Mater. Phys..

[cit20] Wu X. J., Yang J. L., Hou J. G., Zhu Q. S. (2004). J. Chem. Phys..

[cit21] Kumar R., Mertiny P., Parashar A. (2016). J. Phys. Chem. C.

[cit22] Falin A., Cai Q. R., Santos E. J. G., Scullion D., Qian D., Zhang R., Yang Z., Huang S. M., Watanabe K., Taniguchi T., Barnett M. R., Chen Y., Ruoff R. S., Li L. H. (2017). Nat. Commun..

[cit23] Mermin N. D. (1968). Phys. Rev..

[cit24] Fasolino A., Los J. H., Katsnelson M. I. (2007). Nat. Mater..

[cit25] Thomas S., Ajith K. M., Chandra S., Valsakumar M. C. (2015). J. Phys.: Condens. Matter.

[cit26] Anees P., Valsakumar M. C., Panigrahi B. K. (2017). Phys. Chem. Chem. Phys..

[cit27] Pan W., Xiao J. L., Zhu J. W., Yu C. X., Zhang G., Ni Z. H., Watanabe K., Taniguchi T., Shi Y., Wang X. R. (2012). Sci. Rep..

[cit28] Bao W. Z., Miao F., Chen Z., Zhang H., Jang W. Y., Dames C., Lau C. N. (2009). Nat. Nanotechnol..

[cit29] Singh V., Sengupta S., Solanki H. S., Dhall R., Allain A., Dhara S., Pant P., Deshmukh M. M. (2010). Nanotechnology.

[cit30] Yoon D., Son Y. W., Cheong H. (2011). Nano Lett..

[cit31] Thomas S., Ajith K. M., Valsakumar M. C. (2017). Superlattices Microstruct..

[cit32] Meyer J. C., Geim A. K., Katsnelson M. I., Novoselov K. S., Booth T. J., Roth S. (2007). Nature.

[cit33] Pozzo M., Alfe D., Lacovig P., Hofmann P., Lizzit S., Baraldi A. (2011). Phys. Rev. Lett..

[cit34] Thomas S., Ajith K. M., Valsakumar M. C. (2016). J. Phys.: Condens. Matter.

[cit35] Thomas S. A., Ajith K. M., Lee S. U., Valsakumar M. C. (2018). RSC Adv..

[cit36] Ding Y., Wang Y. L. (2014). J. Phys. Chem. C.

[cit37] Sun H., Mukherjee S., Singh C. V. (2016). Phys. Chem. Chem. Phys..

[cit38] Yang Y. L., Xu X. M. (2012). Comput. Mater. Sci..

[cit39] Rajasekaran G., Parashar A. (2016). RSC Adv..

[cit40] Mortazavi B., Ahzi S. (2013). Carbon.

[cit41] Zhao S. J., Xue J. M. (2013). J. Phys. D: Appl. Phys..

[cit42] Zhang P., Ma L. L., Fan F. F., Zeng Z., Peng C., Loya P. E., Liu Z., Gong Y. J., Zhang J. N., Zhang X. X., Ajayan P. M., Zhu T., Lou J. (2014). Nat. Commun..

[cit43] Wei Y. J., Wu J. T., Yin H. Q., Shi X. H., Yang R. G., Dresselhaus M. (2012). Nat. Mater..

[cit44] Juneja A., Rajasekaran G. (2018). Phys. Chem. Chem. Phys..

[cit45] Rahaman O., Mortazavi B., Dianat A., Cuniberti G., Rabczuk T. (2017). Nanotechnology.

[cit46] Thomas S., Ajith K. M., Valsakumar M. C. (2017). Mater. Res. Express.

[cit47] Kumar R., Rajasekaran G., Parashar A. (2016). Nanotechnology.

[cit48] Pereira L. F. C., Mortazavi B., Makaremi M., Rabczuk T. (2016). RSC Adv..

