# Supplementary material for: Atomistic insights into the anisotropic mechanical properties and role of ripples on the thermal expansion of h-BCN monolayers
Source: RSC Adv. 2019 Jan 9;9(3):1238–46. doi: 10.1039/c8ra08076c (PMC9059568; doi:10.1039/c8ra08076c)
Supplement: RA-009-C8RA08076C-s001 [file RA-009-C8RA08076C-s001.pdf]

Electronic Supplementary Material (ESI) for RSC Advances.
This journal is © The Royal Society of Chemistry 2018

Supporting Information

Atomistic Insights into the Anisotropic Mechanical Properties and Role of Ripples on the Thermal Expansion of h-BCN Monolayer

Siby Thomas[1] and Sang Uck Lee[1,2*]*

[1]*Department of Bionano Technology, Hanyang University ERICA, Ansan, Republic of Korea*

[2]*Department of Chemical and Molecular Engineering, Hanyang University ERICA, Ansan, Republic of Korea*

*Corresponding authors Email: siby@cms.hanyang.ac.kr (Siby Thomas)
sulee@hanyang.ac.kr (Sang Uck Lee)

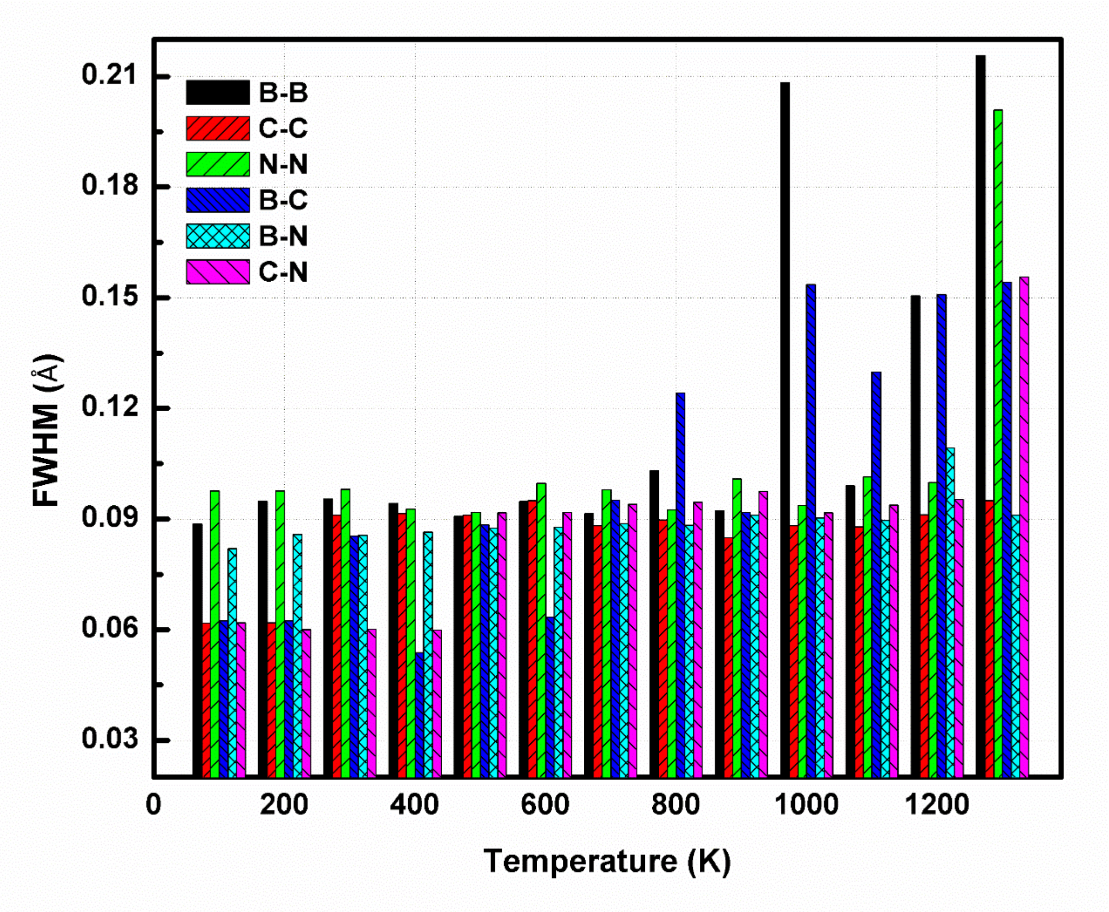

Figure S1. The calculated FWHM of h-BCN monolayer in for different atomic configuration using the optimized Tersoff potential.

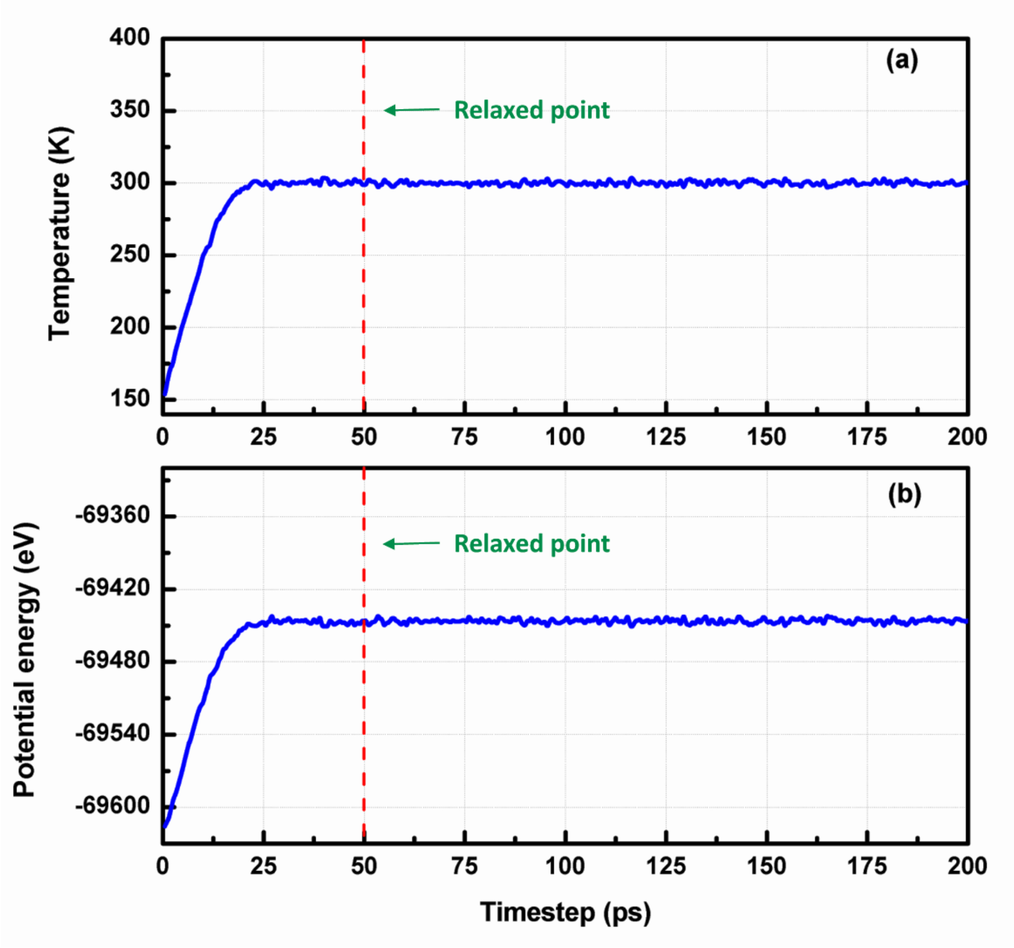

Figure S2. Change in temperature and potential energy of the h-BCN system during MD relaxation simulation. Variation in (a) temperature and (b) total potential energy with respect to the relaxation time.

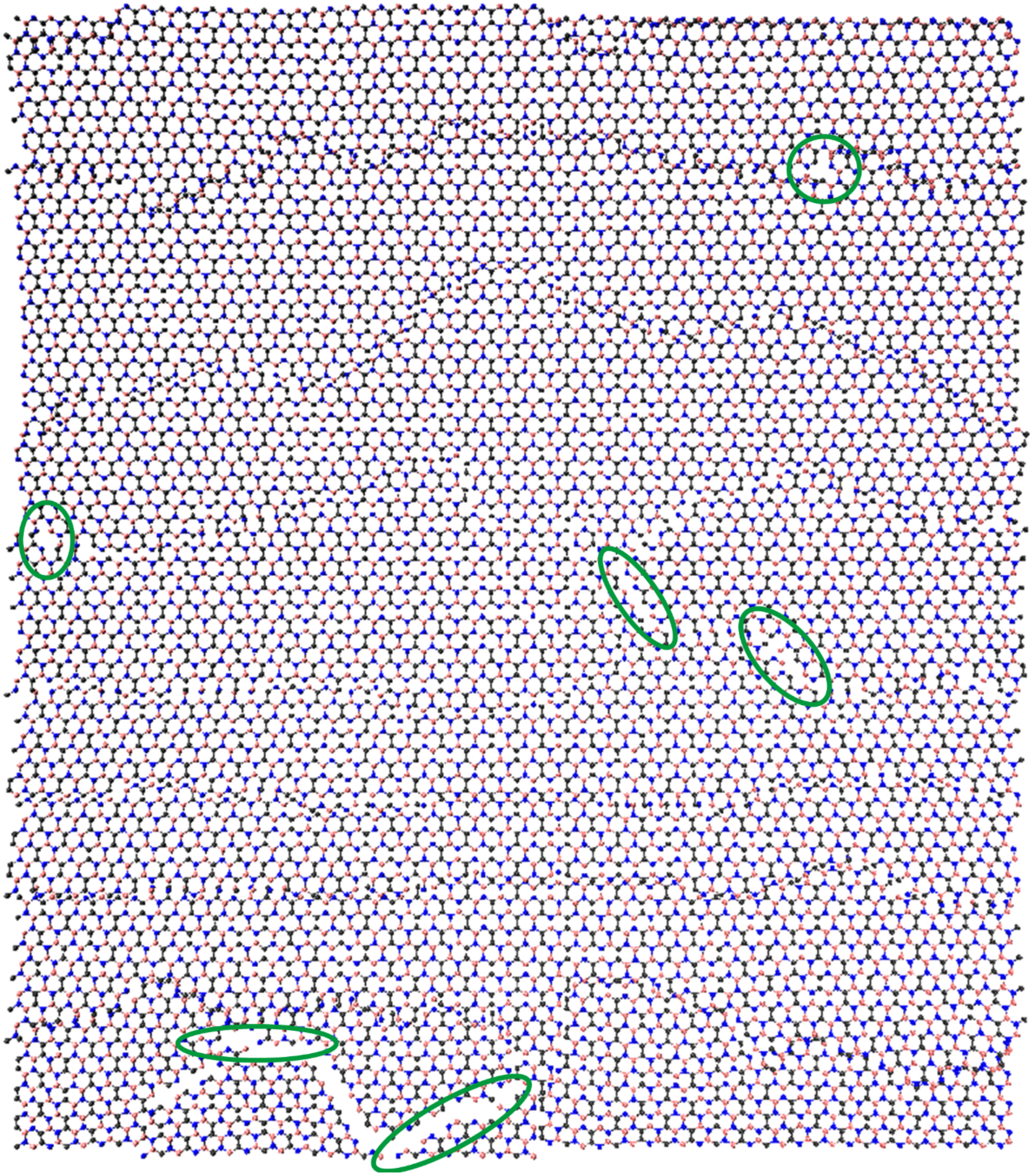

Figure S3. Illustration of the initial stages of failure in h-BCN sheet at 100 K. The green oval shape shows the bond breaking, crack propagation and also the formation of vacancy type defects in the sheet due to the deformation.

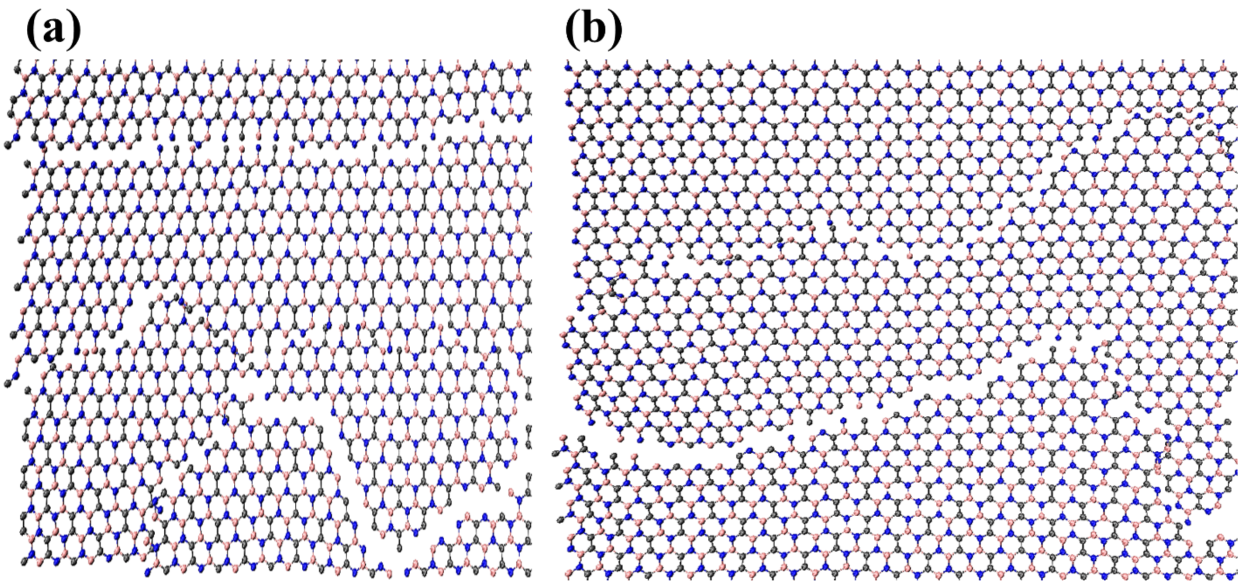

Figure S4. Illustration of the initial stages of failure in h-BCN sheet at 100 K along the (a) armchair and (b) zigzag orientations.
